# Effects of Intrauterine Isoproterenol Administration on Ovarian Follicular Development in Cows

**DOI:** 10.1002/vms3.70198

**Published:** 2025-01-16

**Authors:** Vefa Tohumcu, Mehmet Cengiz, A. Hayirli, K. Altinkaynak, Emre Arslanbas, Alper Yasin Ciplak, S. Aydın, Omercan Alat

**Affiliations:** ^1^ Department of Obstetrics and Gynecology Faculty of Veterinary Medicine Ataturk University Erzurum Turkey; ^2^ Deparment of Obstetrics and Gynecology, Faculty of Veterinary Medicine Mugla Sitki Kocman University Mugla Turkey; ^3^ Department of Animal Nutrition and Nutritional Disorders Faculty of Veterinary Medicine Ataturk University Erzurum Turkey; ^4^ Department of Biochemistry University of Health Sciences Erzurum Regional Training and Research Hospital Erzurum Turkey; ^5^ Department of Pharmacology and Toxicology Faculty of Veterinary Medicine Aksaray University Aksaray Turkey; ^6^ Department of Biochemistry Faculty of Veterinary Medicine Erzurum Turkey

**Keywords:** follicle development, isoproterenol, ovary, vasodilatation

## Abstract

**Background:**

Isoproterenol (ISO) is a nonselective beta‐adrenergic receptor agonist known for its vasodilatory effects. This experiment aims to investigate whether intrauterine ISO administration could alter vascular indices and follicular development in postpartum Holstein cows.

**Objectives:**

The objectives are to evaluate the effects of intrauterine ISO administration on vascular changes and its impact on follicular development compared to placebo groups.

**Study Design:**

This randomized controlled study was conducted on 36 Holstein cows selected based on their health status, including only those free from reproductive, metabolic and infectious disorders.

**Methods:**

The cows (*n* = 36) were divided into two groups as control received distilled water alone (CON, *n* = 18) and experiment received 4 mg ISO in 40 mL distilled water (ISO, *n* = 18) and four subgroups as CON‐I (*n* = 9), CON‐II (*n* = 9), ISO‐I (*n* = 9) and ISO‐II (*n* = 9) according to days of intrauterine administration (I or II represents to 1 or 2 days after ovulation, respectively). Uterine and ovarian artery blood flows were assessed before and after administration by Doppler ultrasonography. Blood samples were collected both before and after administration (on Day 1 or 2) and on Days 3, 6 and 9 post‐ovulation for hormonal analysis. Antral follicle count (AFC) was recorded on the blood sampling days. Data were analysed via mixed model ANOVA.

**Results:**

Intrauterine ISO administration significantly increased the pulse rate (PR) in the ovaries (89.4 vs. 65.5 bpm, *p* < 0.0001) and uterus (90.6 vs. 64.2 bpm, *p* < 0.0001). Early AFC (1–2.9 mm) decreased, whereas small AFC (3–4.9 mm) increased in the ISO groups. The weighted average antral follicle size (WAAFS) significantly increased in the ISO group but remained unchanged in the controls. Hormonal analysis revealed elevated levels of FSH (626 vs. 468 mIU/mL), AMH (61.3 vs. 46.4 ng/L), E2 (138 vs. 122 ng/L), P4 (15.3 vs. 10.6 ng/mL), IGF‐1 (62.6 vs. 25.1 ng/mL) and IGFBP‐3 (28.4 vs. 16.5 ng/mL) in the ISO groups (*p* < 0.0001).

**Conclusions:**

The findings indicate that intrauterine administration of ISO on Day 1 post‐ovulation could be a promising ‘adjunct technique’ for future research focussed on minimizing dependence on exogenous hormones or improving the sensitivity of follicles to endogenous hormonal signals, thereby potentially enhancing oocyte yield.

## Graphical abstract

Distilled water containing isoproterenol was administered intrauterine route on Day 1 or 2 post‐ovulation. A total of 40 mL was infused (20 mL per uterine horn). This treatment increased the pulsation rate in the arteria uterina media and arteria ovarica, leading to an elevated small antral follicle count (3–4.9 mm).

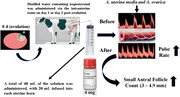

## 1 Introduction

Isoproterenol (ISO) is a nonselective beta‐adrenergic receptor agonist widely recognized for its potent inotropic and chronotropic effects on the cardiovascular system (Goyal et al. 2011). It is primarily metabolized in the liver by catechol‐*O*‐methyltransferase (Nambiar and Chalappurath 2019) and excreted in urine as sulphate conjugates (Reyes et al. 1993; Szefler and Acara 1979). ISO has been widely employed in physiological and pharmacological studies to induce myocardial infarction via subcutaneous or intraperitoneal administration (Benjamin et al. 1989; Goyal et al. 2011; Khan et al. 2022). Despite its extensive use, the effects of intrauterine ISO administration remain undocumented in previous studies.

Ovarian follicular development is a dynamic and continuous process that begins during foetal life and persists throughout a cow's lifespan (Pfeiffer, Jury, and Larson 2014). This process is regulated by periodic surges of follicle‐stimulating hormone (FSH), occurring approximately every 8–10 days. These surges precede the initiation of follicular waves, which gradually decline as the wave progresses and remain suppressed until ovulation or the regression of the dominant follicle (Ireland et al. 2000; Kaneko et al. 1995; Sakaguchi et al. 2019). Typically, 2 or 3 follicular waves (occasionally 1–4) occur during the 21‐day oestrous cycle. In cows with two follicular waves, the waves typically emerge on Days 2–4 and 9–14 (Bodensteiner et al. 1996; Knopf et al. 1989).

Anti‐Müllerian hormone (AMH), produced by granulosa cells in growing follicles, serves as a key biomarker for evaluating ovarian reserve (Ireland et al. 2000; Rico et al. 2009). FSH, secreted from the anterior pituitary gland, primarily drives the growth of gonadotropin‐sensitive follicles, whereas AMH regulates follicular development during the gonadotropin‐independent stage (Koca et al. 2024). In addition, oestrogen (E2) and progesterone (P4) play roles in modulating follicular wave progression and establishing follicular dominance. Insulin‐like growth Factor 1 (IGF‐1) and its binding protein, IGFBP‐3, further support follicular growth and maturation by enhancing gonadotropin signalling. Moreover, metabolic markers such as beta‐hydroxybutyrate (BHBA) and cholesterol (CHOL) provide valuable insights into maternal energy reserves and their influence on reproductive health (Grimard et al. 2013; Wathes 2012).

Doppler indices, such as resistance index (RI) and pulsatility index (PI), are widely used to evaluate vascular resistance and blood flow, offering key insights into uterine and ovarian perfusion (Abouelela et al. 2021; Diaz et al. 2019; Hassan et al. 2017; Rawy et al. 2018). These indices are essential for understanding haemodynamic changes in the reproductive organs. Pulse rate (PR) reflects blood flow and vascular activity, while time‐averaged mean velocity (TAMEAN) and time‐averaged maximum velocity (TAMAX) assess blood flow velocities, crucial for evaluating vascular adaptations in uterine and ovarian arteries (Benjamin et al. 1989; Beltrame et al. 2017; Bernstein and Crane 1991; Elmetwally, Rohn, and Meinecke‐Tillmann 2016; Rawy et al. 2018). Artery diameter indicates vascular compliance and adaptability, offering a comprehensive view of reproductive blood flow dynamics (Abouelela et al. 2021; Diaz et al. 2019).

Previous studies have documented the effects of parenteral administration of various drugs (l‐arginine, oestradiol benzoate, eCG, FSH, etc.) on ovarian and uterine arterial blood flow, as well as on follicular development (Abdelnaby, Abo El‐Maaty, and El‐Badry 2020; Abdelnaby et al. 2023; El‐Sherbiny et al. 2022; Rawy et al. 2018). However, the present study represents the first known report investigating the effects of ISO on follicular development (Web of Science keywords: cow, ovary, uterus, isoproterenol). The aims of this study are to evaluate the impact of intrauterine ISO administration on ovarian blood flow and to investigate its potential role in increasing the number of gonadotropin‐sensitive antral follicles over time.

## 2 Materials and Methods

### 2.1 Animal Management

This experiment was conducted on a private dairy farm located in Aşkale, Erzurum, Turkey. The study involved 36 Holstein cows in their second lactation, with an average body weight of 500 kg, an average daily milk yield of 32 kg and a body condition score (BCS) of 2.75. All cows were confirmed to be free of gynaecological and metabolic disorders and were kept under standard management and nutritional conditions. The animals were provided with a mixed ration and concentrates, with feed and water offered ad libitum. This prospective study was approved by the Atatürk University Local Board of Ethics Committee (158/2020).

### 2.2 Adjustment of ISO Dosage

**FIGURE 1:**
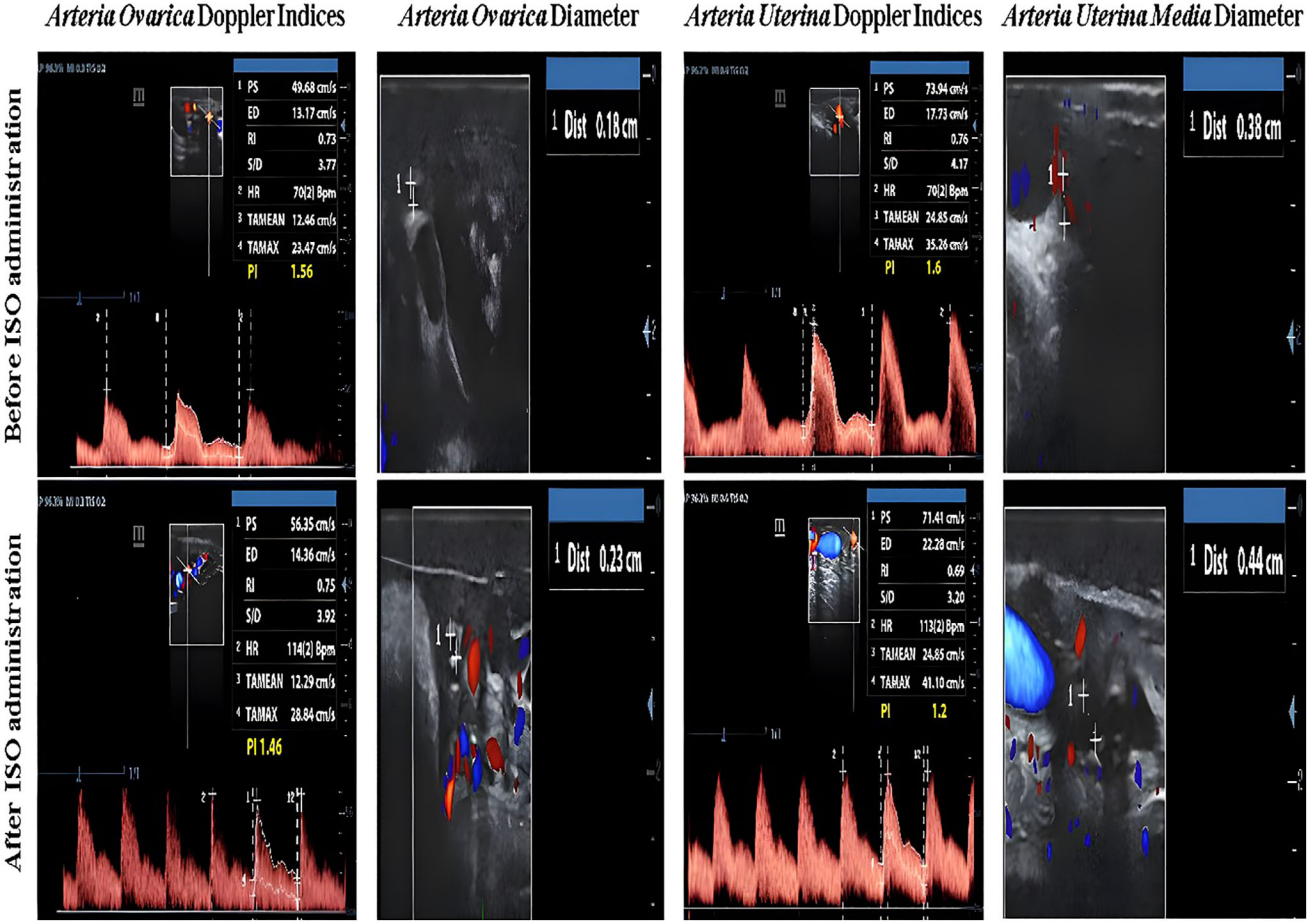
Alterations in spectral images and artery diameters in ovarium (arteria ovarica) and uterus (arteria uterine media) before and after administration of isoproterenol.

ISO (100 mg powder, #I6504; (−)‐isoproterenol hydrochloride; Sigma‐Aldrich, St. Louis, MO) was dissolved in sterile distilled water following the protocol described by Valdivieso et al. (2018). A preliminary study was conducted to determine the optimal dosage, starting with the least harmful doses previously reported in rats (Gyongyosi et al. 2019) and calves (Bruckmaier and Blum 1992). Dosages of 2, 4 and 8 mg ISO were administered by intrauterine route at 1‐week intervals. The cows were monitored for vital signs, including body temperature, heart rate and respiratory rate, as well as for general behaviour, such as alertness and environmental interest. The 4‐mg dose elicited consistent haemodynamic responses without any adverse effects. At this dose, arterial colour flow increased, Doppler waveform frequencies rose and palpable arterial pulsations were observed within 30 min, subsiding by 60 min. These transient changes highlight the temporary nature of the haemodynamic response (Figure 1).

### 2.3 Experimental Design

**FIGURE 2:**
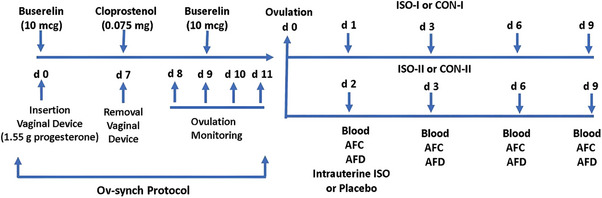
The outline of the experimental protocol. AFC, antral follicle count; AFD, antral follicle diameter; D1, first day after ovulation; D2, second day after ovulation; D3, third day after ovulation; D6, sixth day after ovulation; D9, ninth day after ovulation.

All cows, beginning 60 days postpartum, underwent the standard 7‐day Ov‐Synch protocol with P4. An intravaginal P4‐releasing device (PRID Delta; Ceva Sante Animale, Libourne, France) was aseptically inserted, followed by an intramuscular injection of 10 mcg buserelin acetate (Receptal; MSD, Unterschleissheim, Germany). After 7 days, the PRID was removed, and 0.075 mg of cloprostenol sodium (Estropur; Bioveta, Ivanovice na Hane, Czech Republic) was administered intramuscularly (Figure 2). Ovulation was monitored via transrectal ultrasound (7.5 MHz linear probe, Z60; Mindray, Jiangsu, China) twice daily (09:00–21:00) from 24 to 96 h after PRID removal. Cows not ovulating within 48 h received an additional intramuscular injection of 10 mcg buserelin. The day of ovulation was recorded as Day 0 (D0). The cows (*n* = 36) were divided into two groups as control received distilled water alone (CON, *n* = 18) and experiment received 4 mg ISO in 40 mL distilled water (ISO, *n* = 18) and four subgroups as CON‐I (*n* = 9), CON‐II (*n* = 9), ISO‐I (*n* = 9) and ISO‐II (*n* = 9) according to days of intrauterine administration (I or II represents to 1 or 2 days after ovulation, respectively) (Figure 2). One cow from the CON‐II group was excluded from analysis due to elevated BHBA levels detected at the end of the study, indicating ketosis. Group assignments considered follicular wave initiation, defined as 2 days post‐ovulation (Bodensteiner et al. 1996; Knopf et al. 1989; Sirois and Fortune 1988).

### 2.4 Intrauterine Administrations

The cows were restrained in a chute, and all procedures were conducted under these conditions. All intrauterine applications were performed following aseptic preparation of the perineal region. The perineum and vulva were cleaned with 0.1% diluted povidone‐iodine solution and dried before infusion. After aseptic preparation, a sterile metal catheter was inserted into the vagina, passed through the cervix and positioned in the uterus. Equal volumes of the solution (20 mL) were then slowly infused into each uterine horn. All intrauterine infusions were performed by the same veterinarian. Cows in the ISO‐I and ISO‐II groups received 40 mL of sterile distilled water containing 4 mg ISO, while cows in the CON‐I and CON‐II groups received only 40 mL of sterile distilled water (Figure 2). An additional 10 mL of air was infused to fully empty the catheter and ensure complete delivery of the solution into the uterus, minimizing the risk of retrograde flow. Pre‐Doppler ultrasonographic evaluations confirmed that the solution reached both uterine horns. Before the procedure, the volume of the solution was carefully calculated to match the average capacity of the uterine horns, reducing the risk of retrograde flow (De Oliveira et al. 2020). Follow‐up ultrasonographic examinations performed 1–2 days post‐infusion confirmed complete absorption of the solution in all cows.

### 2.5 Measurement of Doppler Indices

Before Doppler ultrasonography (USG), high caudal epidural anaesthesia was administered at the intercoccygeal space (S5–Co1) with 60‐mg lidocaine hydrochloride (Adokain; Sanovel, Istanbul, Turkey) to improve image quality and minimize artefacts from intestinal and tail movements. Doppler measurements were performed using a colour Doppler USG device (Z60; Mindray) with a linear probe at 7.5 MHz frequency. The same operator conducted measurements on each cow for at least 15 min, both before and after intrauterine administration. All procedures were performed 2 h after milking, with the cows restrained in a standing position.

Blood flow indices were measured using the pulsed wave Doppler USG function. To ensure consistency, Doppler USG settings, including flow direction (60°) and colour gain (∼42), were standardized. A high‐pass filter at 100 Hz was used to eliminate motion artefacts, and the pulse repetition frequency (PRF) was set at 3.6 kHz. Arteries were located using colour mapping, and the sample gate cursor was centred within the vessel to record pulsed Doppler waves. The arteria ovarica and arteria uterina media on both sides were examined using the methods described by Hassan et al. (2017) and Diaz et al. (2019). Measurements were taken from the largest artery in the pedicle, and mean values from both sides were calculated. The sample gate cursor was set at 0.5 mm. Doppler indices, including RI, PI, PR, TAMEAN, TAMAX and artery diameter (AD), were recorded before and after intrauterine administration. To prevent thermal effects from the PRF, pulsed Doppler examinations were limited to 30 s, with a 1‐min pause between each session.

### 2.6 Evaluation of Follicular Development

Follicular development was assessed using a B‐mode real‐time USG device with a 7.5‐MHz linear probe (Z60; Mindray). Three video recordings (≥ 5 s each) were captured per ovary at a 180° angle using the end‐to‐end method (Burns et al. 2005; Cardoso et al. 2018). Evaluations were conducted by a single operator on D1 or D2 after ovulation (D0) and extended to D3, D6 and D9 within the same time frame (17:00–18:00) (Figure 2). Antral follicles were categorized by diameter into antral follicle count (AFC) groups: early (1–2.9 mm), small (3–4.9 mm), intermediate (5–6.9 mm), large (7–8.9 mm) and preovulatory (≥ 9 mm), along with their weighted average follicle size (WAAFS) (Garcia‐Guerra et al. 2015; Lussier, Matton, and Dufour 1987; Mihm and Bleach 2003; Rico et al. 2009).

### 2.7 Collection of Blood Samples and Serum Analysis

Blood samples were collected from the coccygeal vein on the specified days (Figure 2), centrifuged at 1200 × *g* at 4°C for 10 min, and the sera were stored at −80°C until analysis. Hormonal and metabolic parameters were measured using bovine‐specific ELISA kits (Bioassay Technology Laboratory, Shanghai, China) according to the manufacturer's protocols. The analysed parameters included FSH (#EA0025BO; sensitivity: 3.72 mIU/mL), AMH (#EA0241BO; sensitivity: 1.52 g/L), E2 (#EA0093BO; sensitivity: 0.51 ng/L), P4 (#EA0008BO; sensitivity: 0.14 ng/mL), IGF‐1 (#E0016BO; sensitivity: 0.53 ng/mL), IGFBP‐3 (#E0017BO; sensitivity: 0.5–200 ng/mL), BHBA (#E0267BO; sensitivity: 4.89 nmol/mL) and CHOL (#E2030BO; sensitivity: 2.42 mg/dL). The intra‐ and inter‐assay CV values were calculated as 10% and 12% for FSH, E2 and P4, and 8% and 10% for AMH, IGF‐1, BHBA and CHOL, ensuring reliable and consistent measurements.

### 2.8 Statistical Analysis

A mixed model ANOVA was used to evaluate the effects of treatment group (ISO‐I, ISO‐II, CON‐I, CON‐II), time (Doppler indices: pre‐ and 30 min post‐administration; hormonal parameters: pre‐administration, 30 min post‐administration, and D3, D6 and D9; AFC: pre‐administration and D3, D6 and D9) and their interaction (Group × Time). Pre‐ and 30‐min post‐administration measurements were analysed as repeated measures. The model included Doppler indices (RI, PI, TAMAX, TAMEAN, AD), hormonal/metabolic parameters (FSH, AMH, E2, P4, IGF‐1, IGFBP‐3, BHBA, CHOL) and total antral follicle count (TAFC) by size (1–2.9 , 3–4.9 , 5–6.9 , 7–8.9 , ≥ 9 mm) and WAAFS. Individual animal ID was treated as a random effect, with the animal as the experimental unit. Data normality was checked using the Shapiro–Wilk and Kolmogorov–Smirnov tests. Non‐significant parameters were sequentially excluded to refine the model. Group differences were tested using Fisher's LSD. A power analysis based on AMH levels estimated a sample size of nine cows per group to achieve 90% power (*α* = 0.05, *β* = 0.10), assuming a 0.60‐unit improvement and 0.50 standard deviation. Calculations were performed with PS—Power and Sample Size Calculations Version 3.1.6 (Vanderbilt University, Nashville, TN). Statistical significance was set at *p* < 0.05, and results are presented as mean ± SEM.

## 3 Results

### 3.1 Doppler Indices

**FIGURE 3:**
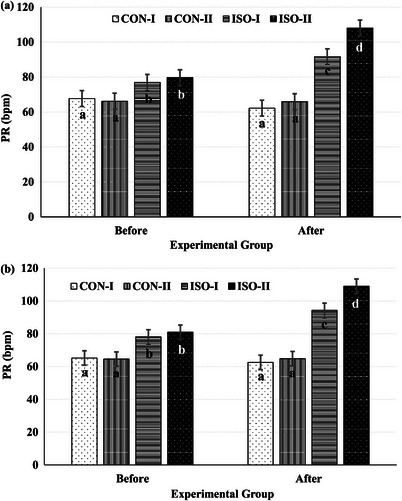
Alterations in pulse rate in ovarium (a) (*p* = 0.0085) and uterus (b) (*p* = 0.0158) over time in response to intrauterine isoproterenol administration (4 mg within 40 mL sterile distilled water) on Day 1 or 2 relative to ovulation. Different letters (a < b < c < d) indicate significant differences between groups at the same time and between different time points (*p* < 0.05). Data are mean ± SEM.

Except for PI and AD, the treatments altered Doppler indices in the ovarium (Table 1). The RI value was the highest when ISO was administered by use of intrauterine administration on D2 relative ovulation. Intrauterine ISO administration increased PR as compared with sterile distilled water (89.4 vs. 65.5 bpm, *p *< 0.0001). However, the ISO administration day did not affect PR. The TAEMEAN and TAMAX values were the highest in the CON‐I group, followed by the ISO‐II group. Both values were greater when ISO was administered by use of intrauterine administration on D2 relative to ovulation than on D1 relative to ovulation. There were no alterations in arteria ovarica Doppler indices in ovarium measured between before and after the treatment except for PR. The PR value increased to a greater extent after administration in the ISO groups than in the CON groups as compared to their values before administration (*p *= 0.0085; Figure 3a). The highest increase was noted in the ISO‐II group.

**TABLE 1:** Thauterine isoproterenol (ISO) administration on Day 1 or 2 relative to ovulation on arteria ovarica Doppler ultrasonography indices in ovarium.

		Response variables					
Group	Time	RI	PI	PR	TAMEAN	TAMAX	AD
CON‐I		0.79 ± 0.04^ab^	1.89 ± 0.08^a^	64.9 ± 2.0^b^	12.6 ± 0.8^a^	24.3 ± 1.3^a^	23.4 ± 0.4^a^
CON‐II		0.75 ± 0.09^b^	2.33 ± 0.54^a^	66.1 ± 2.8^b^	10.6 ± 0.6^ab^	21.4 ± 1.0^ab^	22.6 ± 0.5^a^
ISO‐I		0.79 ± 0.09^ab^	1.82 ± 0.13^a^	84.7 ± 3.7^a^	9.13 ± 0.65^b^	19.7 ± 1.1^b^	24.1 ± 1.5^a^
ISO‐II		0.82 ± 0.04^a^	2.32 ± 0.41^a^	94.0 ± 6.6^a^	11.4 ± 0.9^a^	23.6 ± 1.6^a^	24.3 ± 1.1^a^
	B	0.78 ± 0.07	2.08 ± 0.26	72.7 ± 1.9	10.5 ± 0.5	21.2 ± 0.8	22.4 ± 0.6
	A	0.80 ± 0.07	2.10 ± 0.22	82.5 ± 4.6	11.5 ± 0.6	23.5 ± 1.0	24.8 ± 0.7

	*p*					
**ANOVA**						
Group	0.0289	0.6094	0.0001	0.0238	0.0488	0.6005
Time	0.3265	0.9818	0.0157	0.2018	0.0872	0.0133
Group × Time	0.2893	0.4590	0.0085	0.4507	0.2457	0.0790

*Note*: Data are least square means ± SEM.

Superscripts ‘a’ and ‘b’ indicate statistically significant differences (*p* < 0.05) between groups within the same parameter, as determined by post hoc analysis.

Abbreviations: A, Doppler measurement 30 min after intrauterine administration; AD, artery diameter (mm); B, Doppler measurement 30 min before intrauterine administration; CON‐I, group administered with 40 mL sterile distilled water on D1 relative to ovulation; CON‐II, group administered with 40 mL sterile distilled water on D2 relative to ovulation; ISO‐I, group administered with 4 mg ISO within 40 mL sterile distilled water on D1 relative to ovulation; ISO‐II, group administered with 4 mg ISO within 40 mL sterile distilled water on D2 relative to ovulation; PI, pulsatility index; PR, pulse rate (bpm); RI, resistance index; TAMAX, time‐averaged maximum velocity (cm/s); TAMEAN, time‐averaged mean velocity (cm/s).

Intrauterine ISO administration significantly increased PR (90.6 vs. 64.2 bpm, *p* < 0.0001) and decreased artery diameter (AD) (40.2 vs. 50.7 mm, *p* < 0.0001) compared to intrauterine administration of sterile distilled water (Table 2). No significant alterations were detected in the other Doppler indices of the uterus in response to the administered treatments. The increase in PR was significantly more pronounced in the ISO groups compared to the CON groups relative to their respective baseline values before administration (*p* = 0.0158; Figure 3b). Among all groups, the most substantial increase in PR was observed in the ISO‐II group.

**TABLE 2:** The effects intrauterine isoproterenol (ISO) administration on Day 1 or 2 relative to ovulation on *A. uterine media* Doppler ultrasonography indices in uterus.

		Response variables					
Group	Time	RI	PI	PR	TAMEAN	TAMAX	AD
CON‐I		0.83 ± 0.01^a^	2.28 ± 0.09^a^	63.8 ± 1.8^b^	17.1 ± 0.8^a^	31.9 ± 1.18^a^	50.6 ± 2.1^a^
CON‐II		1.10 ± 0.28^a^	2.13 ± 0.11^a^	64.7 ± 2.5^b^	16.5 ± 1.3^a^	31.5 ± 1.91^ab^	50.8 ± 1.8^a^
ISO‐I		0.81 ± 0.02^a^	1.94 ± 0.13^a^	86.2 ± 3.7^a^	12.6 ± 1.4^b^	26.6 ± 1.89^b^	39.7 ± 2.4^b^
ISO‐II		0.84 ± 0.01^a^	2.02 ± 0.10^a^	94.9 ± 6.6^a^	16.0 ± 1.5^ab^	30.5 ± 2.36^ab^	40.8 ± 1.7^b^
	B	0.95 ± 0.13	2.12 ± 0.08	72.4 ± 1.8	15.1 ± 1.0	28.8 ± 1.48	45.0 ± 1.7
	A	0.82 ± 0.01	2.06 ± 0.07	83.2 ± 4.7	16.0 ± 0.8	31.5 ± 1.17	45.6 ± 1.6

	*p*					
**ANOVA**						
Group	0.3687	0.1529	0.0001	0.0695	0.2062	0.0001
Time	0.2701	0.6222	0.0068	0.5643	0.1985	0.7820
Group × Time	0.4189	0.6080	0.0158	0.7589	0.3194	0.9306

*Note*: Data are least square means ± SEM.

Superscripts ‘a’ and ‘b’ indicate statistically significant differences (*p* < 0.05) between groups within the same parameter, as determined by post hoc analysis.

Abbreviations: A, Doppler measurement 30 min after intrauterine administration; AD, artery diameter (mm); B, Doppler measurement 30 min before intrauterine administration; CON‐I, group administered with 40 mL sterile distilled water on D1 relative to ovulation; CON‐II, group administered with 40 mL sterile distilled water on D2 relative to ovulation; ISO‐I, group administered with 4 mg ISO within 40 mL sterile distilled water on D1 relative to ovulation; ISO‐II, group administered with 4 mg ISO within 40 mL sterile distilled water on D2 relative to ovulation; PI, pulsatility index; PR, pulse rate (bpm); RI, resistance index; TAMAX, time‐averaged maximum velocity (cm/s); TAMEAN, time‐averaged mean velocity (cm/s).

### 3.2 Sizes and Distributions of Antral Follicles

**FIGURE 4:**
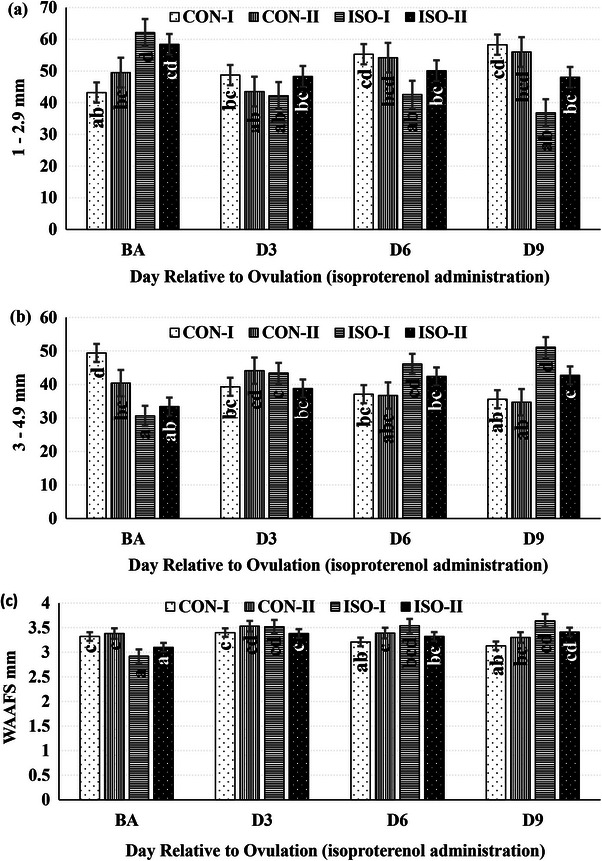
Alterations in percentages of 1–2.9 (a) (*p* < 0.0001) and 3–4.9 (b) (*p* < 0.0001) mm‐antral follicle count and weighted average antral follicle size (WAAFS) (c) (*p* = 0.0073) over time in response to intrauterine isoproterenol (ISO) administration (4 mg within 40 mL sterile distilled water) on Day 1 or 2 relative to ovulation. BA, 30 min before intrauterine ISO administration; D3, D6 and D9, 3, 6 and 9 days after intrauterine ISO administration. Different letters (a < b < c < d) indicate significant differences between groups at the same time and between different time points (*p* < 0.05). Data are mean ± SEM.

TAFC, early (1–2.9 mm), small (3–4.9 mm), intermediate (5–6.9 mm), large (7–8.9 mm) and preovulatory (≥ 9 mm) AFC, as well as the WAAFS, showed no significant differences between groups (Table 3). However, the percentage of intermediate‐sized antral follicles exhibited some sensitivity to intrauterine ISO administration, particularly on D1 relative to ovulation. The preovulatory antral follicle percentage was significantly higher in the ISO‐II group compared to others (*p* < 0.05). During the post‐ovulation period, the percentage of early antral follicles increased in the CON groups but decreased in the ISO groups (*p* < 0.0001; Figure 4a). Conversely, both the percentage of small antral follicles (*p* < 0.0001; Figure 4b) and the WAAFS (*p* = 0.0073; Figure 4c) decreased in the CON groups, while they increased in the ISO groups. Notably, the increases in these parameters were more pronounced in the ISO‐I group compared to the ISO‐II group.

**TABLE 3:** The effects intrauterine isoproterenol (ISO) administration on D1 or D2 relative to ovulation on total antral follicle count (TAFC, *n*), percentage of antral follicles by size (%) and weighted average of antral follicle size (WAAFS, mm).

		Response variables						
Group	Time	TAFC	1–2.9 mm	3–4.9 mm	5–6.9 mm	7–8.9 mm	≥ 9 mm	WAAFS
CON‐I		74.3 ± 3.2^a^	51.4 ± 1.8^a^	40.3 ± 1.6^a^	5.11 ± 0.67^b^	1.79 ± 0.38^a^	1.43 ± 0.25^b^	3.27 ± 0.05^a^
CON‐II		66.8 ± 3.4^a^	50.8 ± 2.4^a^	39.0 ± 2.0^a^	5.97 ± 0.69^ab^	1.54 ± 0.26^a^	2.71 ± 0.36^a^	3.40 ± 0.05^a^
ISO‐I		67.1 ± 2.9^a^	45.9 ± 2.6^a^	42.8 ± 1.9^a^	7.68 ± 0.95^a^	2.15 ± 0.35^a^	1.42 ± 0.25^b^	3.41 ± 0.08^a^
ISO‐II		72.8 ± 4.1^a^	51.2 ± 1.7^a^	39.4 ± 1.5^a^	5.84 ± 0.73^ab^	1.39 ± 0.32^a^	2.22 ± 0.28^a^	3.30 ± 0.05^a^
	B	68.4 ± 3.8^a^	53.4 ± 2.3^a^	38.4 ± 2.0^a^	5.88 ± 0.76^b^	1.48 ± 0.29^b^	0.85 ± 0.26^b^	3.17 ± 0.06^b^
	D3	68.9 ± 3.6^a^	45.7 ± 1.8^b^	41.3 ± 1.4^a^	8.38 ± 0.87^a^	2.40 ± 0.36^a^	2.14 ± 0.29^a^	3.46 ± 0.05^a^
	D6	70.7 ± 3.3^a^	50.5 ± 2.2^ab^	40.7 ± 1.9^a^	4.73 ± 0.56^b^	1.67 ± 0.35^ab^	2.45 ± 0.30^a^	3.36 ± 0.05^a^
	D9	73.4 ± 3.1^a^	49.6 ± 2.4^ab^	41.2 ± 1.8^a^	5.62 ± 0.80^b^	1.34 ± 0.33^b^	2.25 ± 0.26^a^	3.37 ± 0.07^a^

	*p*						
**ANOVA**							
Group	0.2955	0.1466	0.3171	0.1012	0.3682	0.0016	0.1934
Time	0.7228	0.0556	0.5602	0.0074	0.1205	0.0001	0.0050
Group × Time	0.4457	0.0001	0.0001	0.8239	0.1912	0.5100	0.0073

*Note*: Data are least square means ± SEM.

Superscripts ‘a’ and ‘b’ indicate statistically significant differences (*p* < 0.05) between groups within the same parameter, as determined by post hoc analysis.

Abbreviations: B, USG measurement 30 min before intrauterine administration; CON‐I, group administered with 40 mL sterile distilled water on D1 relative to ovulation; CON‐II, group administered with 40 mL sterile distilled water on D2 relative to ovulation; D3, D6 and D9, USG measurements 3, 6 and 9 days after intrauterine administration; ISO‐I, group administered with 4 mg ISO within 40 mL sterile distilled water on D1 relative to ovulation; ISO‐II, group administered with 4 mg ISO within 40 mL sterile distilled water on D2 relative to ovulation.

### 3.3 Hormonal and Metabolic Profile

Intrauterine ISO administration was associated with increases in serum FSH (626 vs. 468 mIU/mL, *p *< 0.0001), AMH (61.3 vs. 46.4 ng/L, *p *< 0.0001), E2 (138 vs. 122 ng/L, *p *< 0.0001), P4 (15.3 vs. 10.6 ng/mL, *p *< 0.0001), IGF‐1 (62.6 vs. 25.1 ng/mL, *p *< 0.0001), IGFBP‐3 (28.4 vs. 16.5 ng/mL, *p* < 0.0001), BHBA (302 vs. 265 nmol/mL, *p* = 0.0067) and CHOL (190 vs. 98.8 mg/dL, *p* < 0.0001) as compared to intrauterine sterile distilled water administration (Table 4). There was neither a time effect nor a treatment by time interaction effect on the hormonal and metabolic profiles.

**TABLE 4:** The effects intrauterine isoproterenol (ISO) administration on Day 1 or 2 relative to ovulation on hormonal and metabolic profile.

		Response variables							
Group	Time	FSH (mIU/mL)	AMH (ng/L)	E2 (ng/L)	P4 (ng/mL)	IGF‐1 (ng/mL)	IGFBP‐3 (ng/mL)	BHBA (nmol/mL)	CHOL (mg/dL)
CON‐I		547 ± 31^b^	37.5 ± 2.9^b^	157 ± 9^a^	11.6 ± 0.6^b^	22.5 ± 1.8^b^	16.0 ± 1.5^b^	195 ± 10^c^	73.7 ± 3.5^c^
CON‐II		388 ± 29^c^	55.4 ± 5.2^a^	87.9 ± 3.5^c^	9.69 ± 0.55^b^	27.7 ± 1.7^b^	17.0 ± 0.9^b^	335 ± 36^ba^	124 ± 10^b^
ISO‐I		601 ± 33^ab^	58.8 ± 4.0^a^	126 ± 7^b^	16.1 ± 1.1^a^	61.7 ± 6.1^a^	27.4 ± 2.3^a^	239 ± 29^bc^	170 ± 14^a^
ISO‐II		652 ± 43^a^	63.8 ± 6.2^a^	151 ± 12^ab^	14.5 ± 1.0^a^	63.4 ± 6.2^a^	29.5 ± 3.3^a^	364 ± 59^a^	209 ± 24^a^
	B	510 ± 44^a^	54.3 ± 4.3^a^	118 ± 10^a^	12.0 ± 0.8^a^	42.0 ± 4.3^a^	22.1 ± 2.4^a^	261 ± 36^a^	138 ± 17^a^
	A	517 ± 40^a^	53.7 ± 5.6^a^	128 ± 12^a^	12.5 ± 1.0^a^	41.0 ± 5.2^a^	21.8 ± 2.8^a^	275 ± 35^a^	141 ± 20^a^
	D3	535 ± 41^a^	55.7 ± 4.8^a^	127 ± 10^a^	12.5 ± 1.1^a^	46.4 ± 6.5^a^	24.1 ± 3.0^a^	339 ± 66^a^	149 ± 18^a^
	D6	595 ± 42^a^	53.1 ± 6.4^a^	143 ± 10^a^	14.1 ± 1.2^a^	46.8 ± 6.9^a^	21.8 ± 2.8^a^	253 ± 33^a^	145 ± 20^a^
	D9	600 ± 44^a^	52.5 ± 6.3^a^	143 ± 11^a^	14.3 ± 1.1^a^	45.1 ± 7.2^a^	23.3 ± 2.6^a^	280 ± 39^a^	151 ± 20^a^

	*p*							
**ANOVA**								
Group	0.0001	0.0011	0.0001	0.0001	0.0001	0.0001	0.0067	0.0001
Time	0.3137	0.9947	0.3285	0.3758	0.9292	0.9653	0.6194	0.9817
Group × Time	0.7865	0.9944	0.8964	0.3846	0.9498	0.9997	0.9934	0.9999

*Note*: Data are least‐square means ± SEM.

Superscripts ‘a’ and ‘b’ indicate statistically significant differences (*p* < 0.05) between groups within the same parameter, as determined by post hoc analysis.

Abbreviations: A, sampling 30 min after intrauterine administration; AMH, anti‐Müllerian hormone; B, sampling 30 min before intrauterine administration; BHBA, beta‐hydroxy butyrate; CHOL, cholesterol; CON‐I, group administered with 40 mL sterile distilled water on D1 relative to ovulation; CON‐II, group administered with 40 mL sterile distilled water on D2 relative to ovulation; D3, D6 and D9, sampling 3, 6, and 9 days after intrauterine administration; E2, oestrogen; FSH, follicle‐stimulating hormone; IGF‐1, insulin‐like growth factor 1; IGFBP‐3, insulin‐like growth factor binding protein 3; ISO‐I, group administered with 4 mg ISO within 40 mL sterile distilled water on D1 relative to ovulation; ISO‐II, group administered with 4 mg ISO within 40 mL sterile distilled water on D2 relative to ovulation; P4, progesterone.

## 4 Discussion

This study evaluated whether intrauterine ISO could induce vasodilation in ovarian and uterine arteries to support follicular development. Like oestradiol benzoate (Rawy et al. 2018), ISO appeared to enhance blood flow, vascular perfusion and hormonal interactions critical for follicular dynamics. However, the lack of significant changes in some Doppler parameters raises questions about the optimal dose and arterial response to ISO.

Doppler USG provides a precise evaluation of regional perfusion and offers valuable insights into organ function. In this study, the absence of differences in RI and PI values between groups in ovarian (Table 1) and uterine (Table 2) arteries may be attributed to the relatively low dosage (4 mg/40 mL) and the limited dilatory capacity of ovarian arteries (Abouelela et al. 2021). The significant increase in PR (Tables 1 and 2; Figure 3) observed following intrauterine ISO administration, compared to the CON groups, aligns with findings from previous studies (Bernstein and Crane 1991; Bruckmaier and Blum 1992). Consistent with earlier research (Honnens et al. 2008), PI values showed no variation among groups. TAMEAN and TAMAX are considered reliable indicators due to the small diameters of ovarian and uterine arteries (Hozumi et al. 2000; Rawy et al. 2018). TAMEAN has been shown to predict delivery time, as pregnancy increases uterine AD, blood flow and TAMAX, which subsequently elevate TAMEAN (Elmetwally, Rohn, and Meinecke‐Tillmann 2016). In this study, TAMEAN and TAMAX values, particularly in the ovaries, varied depending on the timing of intrauterine ISO administration relative to ovulation. Unexpectedly, the highest TAMEAN and TAMAX values were observed in the CON‐I group, which was attributed to individual variations observed before treatment. AD values in the ovaries tended to increase over time in cows treated with intrauterine ISO compared to those administered sterile distilled water (Table 1). However, uterine artery diameters were larger in the CON groups than in the ISO groups. Abouelela et al. (2021) reported a 2 mm increase in ovarian AD in pregnant cows from D7 to D31 of gestation, compared to a 1 mm increase in non‐pregnant cows. This difference may partly be explained by the larger pre‐treatment uterine AD observed in the CON groups compared to the ISO groups. Except for PR, other Doppler indices in this study showed inconsistencies with findings in the existing literature (Beltrame et al. 2017; Hassan et al. 2020).

Intrauterine ISO administration did not affect TAFC (Table 3), so the analysis focussed on percentage changes. Increased embryo yield in cows with high AFC is associated with improved blood flow, which supports follicular development. In dairy cows, high AFC is linked to elevated endothelial nitric oxide synthase protein levels and increased follicular vascularization (Tessaro et al. 2011). However, Holstein cows with high AFC exhibit reduced blood flow to preovulatory follicles (Bonato et al. 2022). In this study, the lack of a treatment × time interaction in Doppler indices indicates that temporary increases in artery diameter and locally active hormones may have a greater role in follicular development than blood flow itself.

Follicles measuring 1–2.9 mm during the preselection phase are gonadotropin‐sensitive but develop independently of gonadotropins (Ireland et al. 2000; Scaramuzzi et al. 2011). These follicles respond to FSH, grow in waves, and persist as a group until deviation (Ireland et al. 2000). In this study, the percentage of 1–2.9 mm follicles decreased in ISO groups but increased in CON groups over time, while the opposite was observed for 3–4.9 mm follicles (Table 3; Figure 3). This suggests that minor Doppler index changes may support early‐stage follicular development in response to intrauterine ISO (Mapletoft et al. 2018; Mihm and Bleach 2003; Scaramuzzi et al. 2011). Gonadotropins stimulate 3–7 mm follicles (Rico et al. 2009). The lack of time‐dependent interactions in 5–6.9, 7–8.9 and ≥ 9 mm follicle percentages (Table 3) likely reflects their reliance on endogenous gonadotropins (Aerts and Bols 2010; Ginther 2016; Mihm and Bleach 2003). WAAFS increased in cows treated with intrauterine ISO but remained stable in those receiving sterile distilled water (Table 3; Figure 3). This indicates that improved regional perfusion enhances follicular development (Hassan et al. 2017; Rocha et al. 2022). Higher WAAFS following intrauterine ISO may also result from elevated serum FSH levels (Table 4) (Adams et al. 1992; Sakaguchi et al. 2019).

FSH influences the development of secondary and tertiary antral follicles (Dierich et al. 1998). Intrauterine ISO improved hormonal profiles that support follicular growth (Table 4). FSH levels reportedly increase in 4–7 mm follicles but decrease during dominant follicle selection (Adams et al. 1992). AFC, a key biomarker of female reproduction, strongly correlates with ovarian reserve and is widely used to assess reproductive performance (Ireland et al. 2008; Moon et al. 2024). AMH, produced by ovarian follicles, is a critical indicator of AFC and follicular development (Juengel et al. 2021). In this study, higher AMH levels in ISO groups were linked to an increased number of 3–4.9‐mm follicles and a decreased number of 1–2.9‐mm follicles (Figure 4; Table 4). In contrast, AMH levels in the CON‐II group were comparable to ISO groups, attributed to initially higher AMH levels in this group. AFC and WAAFS remained stable in CON groups but significantly increased in ISO groups between BA and D9 (Figure 4; Table 4).

The corpus luteum (CL) is a reliable marker of ovarian activity, with P4 secreted by CL essential for embryo development (Pohler et al. 2012). Godkin, Black, and Duby (1977) showed that ISO stimulates cAMP synthesis in luteal cells, increasing P4 production. Similarly, ISO enhanced P4 production in CL in this study (Table 4). P4 is also synthesized to develop follicles and support their function. The E2/P4 ratio favours E2 in dominant follicles but shifts toward P4 in subordinate ones (Assey et al. 1994). Elevated E2 levels in ISO groups likely result from ISO's effect on AFC. Increased E2 after ISO may also enhance blood flow, supporting follicular development (Rawy et al. 2018). Thus, ISO likely increases AFC, leading to elevated E2 levels, which sustain higher blood flow and promote follicular growth.

Circulating IGF‐1 levels positively correlate with fertility (Molina‐Coto et al. 2020). IGF‐1, linked to follicular development, promotes steroid production in response to gonadotropins, explaining higher steroid and FSH levels in ISO groups (Table 4) (Echternkamp et al. 1994). Elevated E2 and IGF‐1 during early follicular development, mediated by FSH, protect recruitment follicles against IGFBP (Silva, Figueiredo, and Van den Hurk 2009). IGFBP‐3 inhibits IGF‐1 and IGF‐2, regulating follicular growth (Cunha‐Filho et al. 2005; Fortune, Rivera, and Yang 2004). High IGFBP‐3 levels, associated with infertility, act as a protective mechanism against excessive follicular growth and were linked to increased 3–4.9 mm AFC and reduced 1–2.9 mm AFC in ISO groups (Grimard et al. 2013). Despite low IGF‐1 levels in CON groups, lower IGFBP‐3 levels in ISO groups align with the hypothesis that 90% of IGFs bind to IGFBP‐3 for transport (Wathes 2012). These changes likely increase demand for maternal reserves, reflected in higher BHBA and CHOL levels in ISO groups (Table 4).

The overall evaluation of the hormonal and metabolic profile revealed that the parameters were higher in the ISO groups compared to the CON groups. The increase in small AFC (3–4.9 mm), classified as transitioning toward gonadotropin sensitivity, in the ISO groups was attributed to hormonal and metabolic elevations rather than blood flow indices (RI, PI, TAMEAN, TAMAX).

## 5 Conclusion

Intrauterine administration of ISO, through its vasodilatory and potential indirect hormonal effects, might enhance blood flow in the ovarian and uterine arteries, thereby supporting the development of small AFCs. However, Doppler indices, except for PR, remained unaffected by intrauterine ISO administration. Notably, ISO administered via the intrauterine route on D1 relative to ovulation increased the number of small AFCs, which are precursors for gonadotropin sensitivity. These findings suggest that intrauterine ISO administration on D1 post‐ovulation could serve as an ‘adjunct technique’ in future studies aimed at reducing dependence on exogenous hormones or enhancing follicular hormone sensitivity, ultimately improving oocyte yield. Furthermore, intrauterine ISO administration could be explored as a complementary approach in anoestrus treatment studies or in pharmacological research focussed on increasing drug concentrations within the uterus.

## Author Contributions

**V. Tohumcu**: conceptualization, methodology, data curation, investigation, supervision, funding acquisition, visualization, writing–original draft, writing–review and editing, validation. **Mehmet Cengiz**: conceptualization, methodology, data curation, investigation, funding acquisition, project administration, writing–original draft, writing–review and editing, validation. **A. Hayirli**: data curation, methodology, formal analysis, software, writing–review and editing, validation. **K. Altinkaynak**: methodology, resources. **Emre Arslanbas**: writing–review and editing, resources, investigation. **Alper Yasin Ciplak**: resources, data curation, visualization. S. **Aydın**: data curation, visualization, resources. **O. Alat**: resources, data curation.

## Ethics Statement

This prospective study was approved by the Atatürk University Local Board of Ethics Committee (158/2020).

## Conflicts of Interest

The authors declare no conflicts of interest.

### Peer Review

The peer review history for this article is available at https://publons.com/publon/10.1002/vms3.70198.

## Data Availability

Dataset and analyses are available from the corresponding author upon reasonable request.
